# Hermite–Hadamard type inequalities for fractional integrals via Green’s function

**DOI:** 10.1186/s13660-018-1751-6

**Published:** 2018-07-04

**Authors:** Muhammad Adil Khan, Arshad Iqbal, Muhammad Suleman, Yu-Ming Chu

**Affiliations:** 1 College of Science, Hunan City University, Yiyang, China; 2 Department of Mathematics, University of Peshawar, Peshawar, Pakistan; 3 Department of Mathematics, Huzhou University, Huzhou, China

**Keywords:** 26D15, Hermite–Hadamard inequality, convex function, Green’s function

## Abstract

In the article, we establish the left Riemann–Liouville fractional Hermite–Hadamard type inequalities and the generalized Hermite–Hadamard type inequalities by using Green’s function and Jensen’s inequality, and present several new Hermite–Hadamard type inequalities for a class of convex as well as monotone functions.

## Introduction

Convexity plays an important role in all the fields of pure and applied mathematics [1–12]. Many remarkable inequalities have been obtained in the literature by using convexity [13–22]. Among the inequalities, the most extensively and intensively attractive inequality in the last decades is the well-known Hermite–Hadamard inequality. This interesting result was obtained by Hermite and Hadamard independently, and it provides an equivalence with the convexity property. This inequality reads as follows: if the function $\psi:[\alpha_{1},\alpha_{2}]\rightarrow \mathbb{R}$ is convex on $[\alpha_{1},\alpha_{2}]$, then

1.1 $$ \psi \biggl(\frac{\alpha_{1}+\alpha_{2}}{2} \biggr)\leq \frac{1}{\alpha_{2}-\alpha_{1}} \int_{\alpha_{1}}^{\alpha_{2}}\psi(x)\,dx \leq \frac{\psi(\alpha_{1})+\psi(\alpha_{2})}{2}. $$

If *ψ* is a concave function, then the inequalities in (1.1) will hold in reverse directions. The Hermite–Hadamard inequality gives an upper as well as lower estimations for the integral mean of any convex function defined on a closed and bounded interval which involves the endpoints and midpoint of the domain of the function. Also (1.1) provides the necessary and sufficient condition for the function to be convex. There are several applications of this inequality in the geometry of Banach spaces and nonlinear analysis [23, 24]. Some peculiar convex functions can be used in (1.1) to obtain classical inequalities for means. For some comprehensive surveys on various generalizations and developments of (1.1), we recommend [25]. Due to the great importance of this inequality, in the recent years many remarkable varieties of generalizations, refinements, extensions and different versions of Hermite–Hadamard inequality for different classes of convexity, such as preinvex, *s*-convex, harmonic convex, $\alpha(x)$-convex, superquadratic, and co-ordinate convex functions, have been studied in the literature. Also there have been a large number of research papers published on this subject, for interested readers we recommend to read the papers [26–37] and some of the references therein.

The following definitions for the left and right side Riemann–Liouville fractional integrals are well known in the literature.

Let $b_{1}, b_{2}\in\mathbb{R}$ with $b_{1}< b_{2}$ and $\psi\in L[b_{1},b_{2}]$. Then the left and right Riemann–Liouville fractional integrals $J_{b_{1}+}^{\alpha}\psi$ and $J_{b_{2}-}^{\alpha}\psi$ of order $\alpha>0$ are defined by

$$ J_{b_{1}+}^{\alpha}\psi(x)=\frac{1}{\Gamma(\alpha)} \int_{b_{1}}^{x}(x-t)^{\alpha-1}\psi(t)\,dt, \quad x>b_{1}, $$

and

$$ J_{b_{2}-}^{\alpha}\psi(x)=\frac{1}{\Gamma(\alpha)} \int_{x}^{b_{2}}(t-x)^{\alpha-1}\psi(t)\,dt, \quad x< b_{2}, $$

respectively, where $\Gamma(\alpha)$ is the gamma function defined by $\Gamma(\alpha)=\int_{0}^{\infty}e^{-t}t^{\alpha-1}\,dt$.

In [38], Sarikaya et al. established the Hermite–Hadamard type inequality for fractional integral as follows.

### Theorem 1.1

*Let* $\psi:[b_{1},b_{2}]\rightarrow\mathbb{R}$ *be a positive function with* $0\leq b_{1}< b_{2}$, $\alpha>0$, *and* $\psi\in L[b_{1},b_{2}]$. *If* *ψ* *is convex on* $[b_{1},b_{2}]$, *then one has*

$$ \psi \biggl(\frac{b_{1}+b_{2}}{2} \biggr)\leq\frac{\Gamma(\alpha+1)}{2(b_{2}-b_{1})^{\alpha}} \bigl[J_{b_{1}+}^{\alpha}\psi(b_{2}) \bigr] +J_{b_{2}-}^{\alpha}\psi(b_{1})\leq\frac{\psi(b_{1})+\psi(b_{2})}{2}. $$

### Remark 1.2

In Theorem 1.1, it is not necessary to suppose that *ψ* is a positive function and $b_{1},b_{2}$ are positive real numbers. From the definition of left and right Riemann–Liouville fractional integrals, we clearly see that $b_{1}$ and $b_{2}$ can be any real numbers such that $b_{1}< b_{2}$.

The main purpose of this paper is to give a new method to derive the left Riemann–Liouville fractional Hermite–Hadamard type inequalities as given in [39]. In this method we use Green’s function and obtain identities for the difference of the left Riemann–Liouville fractional Hermite–Hadamard inequality, and then we prove that these identities are non-negative. As a consequence, these inequalities provide the generalized Hermite–Hadamard inequality. Also, by using these identities for the class of convex, concave, and monotone functions, we obtain new Hermite–Hadamard type inequalities.

## Main results

Let $b_{1}< b_{2}$. Then the following four new Green’s functions $G_{i}: [b_{1}, b_{2}]\times [b_{1}, b_{2}]\mapsto \mathbb{R}$ $(i=1, 2, 3, 4)$ are defined by Mehmood et al. in [40]:

2.1 $$\begin{aligned} &G_{1}(\lambda, \mu)= \textstyle\begin{cases} b_{1}-\mu , \quad b_{1}\leq \mu\leq\lambda,\\ b_{1}-\lambda , \quad \lambda\leq \mu\leq b_{2}, \end{cases}\displaystyle \end{aligned}$$

2.2 $$\begin{aligned} &G_{2}(\lambda, \mu)= \textstyle\begin{cases} \lambda-b_{2}, \quad b_{1}\leq \mu\leq\lambda,\\ \mu-b_{2}, \quad \lambda\leq \mu\leq b_{2}, \end{cases}\displaystyle \end{aligned}$$

2.3 $$\begin{aligned} &G_{3}(\lambda, \mu)= \textstyle\begin{cases} \lambda-b_{1}, \quad b_{1}\leq \mu\leq\lambda,\\ \mu-b_{1}, \quad \lambda\leq \mu\leq b_{2}, \end{cases}\displaystyle \end{aligned}$$

2.4 $$\begin{aligned} &G_{4}(\lambda, \mu)= \textstyle\begin{cases} b_{2}-\mu, \quad b_{1}\leq \mu\leq\lambda,\\ b_{2}-\lambda, \quad \lambda\leq \mu\leq b_{2}. \end{cases}\displaystyle \end{aligned}$$

In [40], the authors established the following Lemma 2.1, which will be used to establish our main results.

### Lemma 2.1

(see [40, Lemma 1])

*Let* $b_{1}< b_{2}$ *and* $G=G_{1}$ *be the Green’s function defined by* (2.1). *Then*

2.5 $$ \psi(x)= \psi(b_{1})+(x-b_{1}) \psi'(b_{2})+ \int_{b_{1}}^{b_{2}} G(x,\mu)\psi''( \mu) \,d\mu $$

*if* $\psi \in C^{2}([b_{1},b_{2}])$.

### Theorem 2.2

*Let* $\psi \in C^{2}([b_{1},b_{2}])$ *be a convex function*. *Then the double inequality*

2.6 $$ \psi \biggl(\frac{\alpha b_{1}+b_{2}}{\alpha+1} \biggr) \leq \frac{\Gamma(\alpha+1)}{(b_{2}-b_{1})^{\alpha}}J_{b_{1}+}^{\alpha} \psi(b_{2}) \leq \frac{\alpha\psi(b_{1})+\psi(b_{2})}{\alpha+1} $$

*holds for any* $\alpha>0$.

### Proof

Substituting $x= \frac{\alpha b_{1}+b_{2}}{\alpha+1}$ in (2.5), we get

2.7 $$ \begin{aligned} & \begin{aligned} \psi \biggl(\frac{\alpha b_{1}+b_{2}}{\alpha+1} \biggr)={}&\psi(b_{1})+ \biggl( \frac{\alpha b_{1}+b_{2}}{\alpha+1}-b_{1} \biggr)\psi'(b_{2}) \\ &{}+ \int_{b_{1}}^{b_{2}} G \biggl(\frac{\alpha b_{1}+b_{2}}{\alpha+1},\mu \biggr) \psi''(\mu) \,d\mu, \end{aligned} \\ &\psi \biggl(\frac{\alpha b_{1}+b_{2}}{\alpha+1} \biggr)=\psi(b_{1})+ \frac{(b_{2}-b_{1})\psi'(b_{2})}{\alpha+1}+ \int_{b_{1}}^{b_{2}} G \biggl(\frac{\alpha b_{1}+b_{2}}{\alpha+1},\mu \biggr) \psi''(\mu) \,d\mu. \end{aligned} $$

Now, multiplying both sides of (2.5) by $\frac{\alpha(b_{2}-x)^{\alpha-1}}{(b_{2}-b_{1})^{\alpha}}$ and integrating, we get

2.8 $$ \begin{aligned} &\frac{\alpha}{(b_{2}-b_{1})^{\alpha}} \int_{b_{1}}^{b_{2}}(b_{2}-x)^{\alpha-1}\psi(x) \,dx \\ &\quad =\frac{\alpha}{(b_{2}-b_{1})^{\alpha}} \biggl[ \int_{b_{1}}^{b_{2}}(b_{2}-x)^{\alpha-1} \psi(b_{1})\,dx+ \int_{b_{1}}^{b_{2}}(b_{2}-x)^{\alpha-1}(x-b_{1}) \psi'(b_{2})\,dx \\ &\qquad {}+ \int_{b_{1}}^{b_{2}} \int_{b_{1}}^{b_{2}}(b_{2}-x)^{\alpha-1}G(x, \mu)\psi''(\mu) \,d\mu \,dx \biggr] \\ &\quad =\frac{\alpha}{(b_{2}-b_{1})^{\alpha}} \biggl[\psi(b_{1})\frac{(b_{2}-b_{1})^{\alpha}}{\alpha}+ \frac{\psi'(b_{2})(b_{2}-b_{1})^{\alpha+1}}{\alpha(\alpha+1)} \\ &\qquad {}+ \int_{b_{1}}^{b_{2}} \int_{b_{1}}^{b_{2}}(b_{2}-x)^{\alpha-1}G(x, \mu)\psi''(\mu) \,d\mu \,dx \biggr], \\ & \begin{aligned} \frac{\Gamma(\alpha+1)}{(b_{2}-b_{1})^{\alpha}}J_{b_{1}+}^{\alpha} \psi(b_{2}) ={}& \psi(b_{1})+\frac{(b_{2}-b_{1})\psi'(b_{2})}{\alpha+1} \\ &{} +\frac{\alpha}{(b_{2}-b_{1})^{\alpha}} \int_{b_{1}}^{b_{2}} \int_{b_{1}}^{b_{2}}(b_{2}-x)^{\alpha-1}G(x, \mu)\psi''(\mu) \,d\mu \,dx. \end{aligned} \end{aligned} $$

Subtracting (2.8) from (2.7), we obtain

2.9 $$ \begin{aligned}[b] &\psi \biggl(\frac{\alpha b_{1}+b_{2}}{\alpha+1} \biggr)- \frac{\Gamma(\alpha+1)}{(b_{2}-b_{1})^{\alpha}}J_{b_{1}+}^{\alpha}\psi(b_{2}) \\ &\quad = \int_{b_{1}}^{b_{2}} \biggl[G \biggl(\frac{\alpha b_{1}+b_{2}}{\alpha+1},\mu \biggr)-\frac{\alpha}{(b_{2}-b_{1})^{\alpha}} \int_{b_{1}}^{b_{2}}(b_{2}-x)^{\alpha-1}G(x, \mu)\,dx \biggr]\psi''(\mu) \,d\mu. \end{aligned} $$

Clearly

2.10 $$ \int_{b_{1}}^{b_{2}}(b_{2}-x)^{\alpha-1}G(x, \mu)\,dx=\frac{1}{\alpha(\alpha+1)} \bigl[(b_{2}-\mu)^{\alpha+1}-(b_{2}-b_{1})^{\alpha+1} \bigr]. $$

Also, since

2.11 $$ G \biggl(\frac{\alpha b_{1}+b_{2}}{\alpha+1},\mu \biggr)= \textstyle\begin{cases} b_{1}-\mu, \quad b_{1}\leq \mu\leq\frac{\alpha b_{1}+b_{2}}{\alpha+1}, \\ \frac{b_{1}-b_{2}}{\alpha+1}, \quad \frac{\alpha b_{1}+b_{2}}{\alpha+1}\leq \mu\leq b_{2}. \end{cases} $$

Therefore, if $b_{1}\leq\mu\leq\frac{\alpha b_{1}+b_{2}}{\alpha+1}$, then from (2.10) and (2.11) we have

$$\begin{aligned} &G \biggl(\frac{\alpha b_{1}+b_{2}}{\alpha+1},\mu \biggr)-\frac{\alpha}{(b_{2}-b_{1})^{\alpha}} \int_{b_{1}}^{b_{2}}(b_{2}-x)^{\alpha-1}G(x, \mu)\,dx \\ &\quad =b_{1}-\mu-\frac{(b_{2}-\mu)^{\alpha+1}-(b_{2}-b_{1})^{\alpha+1}}{(b_{2}-b_{1})^{\alpha}(\alpha+1)}. \end{aligned}$$

Now, let

$$ f(\mu)=b_{1}-\mu-\frac{(b_{2}-\mu)^{\alpha+1}-(b_{2}-b_{1})^{\alpha+1}}{(b_{2}-b_{1})^{\alpha}(\alpha+1)}. $$

Then

$$ f'(\mu)=-1 + \frac{(b_{2}-\mu)^{\alpha}}{(b_{2}-b_{1})^{\alpha}} \leq 0, $$

which shows that *f* is decreasing and $f(b_{1})\leq 0$, hence $f(\mu)\leq 0$ for all $\mu\in[b_{1},\frac{\alpha b_{1}+b_{2}}{\alpha+1}]$, i.e.,

2.12 $$ G \biggl(\frac{\alpha b_{1}+b_{2}}{\alpha+1},\mu \biggr)-\frac{\alpha}{(b_{2}-b_{1})^{\alpha}} \int_{b_{1}}^{b_{2}}(b_{2}-x)^{\alpha-1}G(x, \mu)\,dx \leq 0. $$

If $\frac{\alpha b_{1}+b_{2}}{\alpha+1}\leq\mu\leq b_{2}$, then making use of (2.10) and (2.11) we get

2.13 $$ \begin{aligned}[b] &G \biggl(\frac{\alpha b_{1}+b_{2}}{\alpha+1},\mu \biggr)- \frac{\alpha}{(b_{2}-b_{1})^{\alpha}} \int_{b_{1}}^{b_{2}}(b_{2}-x)^{\alpha-1}G(x, \mu)\,dx \\ &\quad =\frac{b_{1}-b_{2}}{\alpha+1}-\frac{\alpha}{(b_{2}-b_{1})^{\alpha}} \biggl[\frac{(b_{2}-\mu)^{\alpha+1}-(b_{2}-b_{1})^{\alpha+1}}{\alpha(\alpha+1)} \biggr] \\ &\quad =-\frac{(b_{2}-b_{1})}{(\alpha+1)}-\frac{(b_{2}-\mu)^{\alpha+1}-(b_{2}-b_{1})^{\alpha+1}}{(b_{2}-b_{1})^{\alpha}(\alpha+1)} \\ &\quad =\frac{-(b_{2}-b_{1})^{\alpha+1}-(b_{2}-\mu)^{\alpha+1}+(b_{2}-b_{1})^{\alpha+1}}{(\alpha+1)(b_{2}-b_{1})^{\alpha}} \\ &\quad =\frac{-(b_{2}-\mu)^{\alpha+1}}{(\alpha+1)(b_{2}-b_{1})^{\alpha}} \leq 0. \end{aligned} $$

Since *ψ* is convex, therefore, $\psi''(\mu)\geq$ 0 and so by using (2.12) and (2.13) in (2.9), we deduce

$$ \psi \biggl(\frac{\alpha b_{1}+b_{2}}{\alpha+1} \biggr)\leq\frac{\Gamma(\alpha+1)}{(b_{2}-b_{1})^{\alpha}}J_{b_{1}+}^{\alpha} \psi(b_{2}), $$

which is the first inequality of (2.6).

Next, we prove the second inequality of (2.6). Let $x=b_{2}$ in (2.5), then we have

$$ \psi(b_{2})=\psi(b_{1})+(b_{2}-b_{1}) \psi'(b_{2})+ \int_{b_{1}}^{b_{2}}G(b_{2},\mu) \psi''(\mu)\,d\mu. $$

Adding $\alpha\psi(b_{1})$ on both sides and then dividing by $(\alpha+1)$, we get

2.14 $$ \frac{\alpha\psi(b_{1})+\psi(b_{2})}{\alpha+1}=\psi(b_{1})+\frac{(b_{2}-b_{1})\psi'(b_{2})}{\alpha+1}+ \frac{1}{\alpha+1} \int_{b_{1}}^{b_{2}}G(b_{2},\mu) \psi''(\mu)\,d\mu. $$

Subtracting (2.8) from (2.14), we obtain

2.15 $$ \begin{aligned} &\frac{\alpha\psi(b_{1})+\psi(b_{2})}{\alpha+1}-\frac{\Gamma(\alpha+1)}{(b_{2}-b_{1})^{\alpha}}J_{b_{1}+}^{\alpha} \psi(b_{2}) \\ &\quad = \int_{b_{1}}^{b_{2}} \biggl[\frac{G(b_{2},\mu)}{\alpha+1}- \frac{\alpha}{(b_{2}-b_{1})^{\alpha}} \int_{b_{1}}^{b_{2}}(b_{2}-x)^{\alpha-1}G(x, \mu)\,dx \biggr]\psi''(\mu)\,d\mu. \end{aligned} $$

Using the Green’s function and (2.10), we obtain

2.16 $$ \begin{aligned}[b] &\frac{G(b_{2},\mu)}{\alpha+1}-\frac{\alpha}{(b_{2}-b_{1})^{\alpha}} \biggl[ \frac{(b_{2}-\mu)^{\alpha+1}-(b_{2}-b_{1})^{\alpha+1}}{\alpha(\alpha+1)} \biggr] \\ &\quad =\frac{b_{1}-\mu}{\alpha+1}-\frac{(b_{2}-\mu)^{\alpha+1}-(b_{2}-b_{1})^{\alpha+1}}{(\alpha+1)(b_{2}-b_{1})^{\alpha}} \\ &\quad =\frac{(b_{1}-\mu)(b_{2}-b_{1})^{\alpha}+(b_{2}-b_{1})^{\alpha+1}-(b_{2}-\mu)^{\alpha+1}}{(\alpha+1)(b_{2}-b_{1})^{\alpha}} \\ &\quad =\frac{(b_{2}-b_{1})^{\alpha}(b_{2}-\mu)-(b_{2}-\mu)^{\alpha+1}}{(\alpha+1)(b_{2}-b_{1})^{\alpha}} \\ &\quad =\frac{(b_{2}-\mu)[(b_{2}-b_{1})^{\alpha}-(b_{2}-\mu)^{\alpha}]}{(\alpha+1)(b_{2}-b_{1})^{\alpha}} \geq 0 \end{aligned} $$

for all $b_{1}\leq\mu\leq b_{2}$.

Now, using the convexity of *ψ* and (2.16) in (2.15), we get

$$ \frac{\alpha\psi(b_{1})+\psi(b_{2})}{\alpha+1}\geq\frac{\Gamma(\alpha+1)}{(b_{2}-b_{1})^{\alpha}}J_{b_{1}+}^{\alpha} \psi(b_{2}), $$

which is the second inequality of (2.6). □

Next, we present new Hermite–Hadamard type inequalities for the class of monotone and convex functions.

### Theorem 2.3

*Let* $\psi \in C^{2}([b_{1},b_{2}])$ *and* $\alpha>0$. *Then the following statements are true*:

(i) *If* $|\psi''|$ *is an increasing function*, *then*
  2.17 $$ \biggl\vert \frac{\alpha\psi(b_{1})+\psi(b_{2})}{\alpha+1}-\frac{\Gamma(\alpha+1)}{(b_{2}-b_{1})^{\alpha}}J_{b_{1}+}^{\alpha} \psi(b_{2}) \biggr\vert \leq\frac{ \vert \psi''(b_{2}) \vert \alpha(b_{2}-b_{1})^{2}}{2(\alpha+1)(\alpha+2)}. $$
(ii) *If* $|\psi''|$ *is a decreasing function*, *then*
  $$ \biggl\vert \frac{\alpha\psi(b_{1})+\psi(b_{2})}{\alpha+1}-\frac{\Gamma(\alpha+1)}{(b_{2}-b_{1})^{\alpha}}J_{b_{1}+}^{\alpha} \psi(b_{2}) \biggr\vert \leq\frac{ \vert \psi''(b_{1}) \vert \alpha(b_{2}-b_{1})^{2}}{2(\alpha+1)(\alpha+2)}. $$
(iii) *If* $|\psi''|$ *is a convex function*, *then*
  $$\begin{aligned} \biggl\vert \frac{\alpha\psi(b_{1})+\psi(b_{2})}{\alpha+1}-\frac{\Gamma(\alpha+1)}{(b_{2}-b_{1})^{\alpha}}J_{b_{1}+}^{\alpha} \psi(b_{2}) \biggr\vert \leq\frac{\max\{ \vert \psi''(b_{1}) \vert , \vert \psi''(b_{2}) \vert \}\alpha(b_{2}-b_{1})^{2}}{2(\alpha+1)(\alpha+2)}. \end{aligned}$$

### Proof

(i) It follows from (2.15) that

2.18 $$ \begin{aligned}[b] & \biggl\vert \frac{\alpha\psi(b_{1})+\psi(b_{2})}{\alpha+1}- \frac{\Gamma(\alpha+1)}{(b_{2}-b_{1})^{\alpha}}J_{b_{1}+}^{\alpha} \psi(b_{2}) \biggr\vert \\ &\quad \leq\frac{1}{(\alpha+1)(b_{2}-b_{1})^{\alpha}} \int_{b_{1}}^{b_{2}} \bigl[(b_{2}-b_{1})^{\alpha}(b_{2}- \mu)-(b_{2}-\mu)^{\alpha+1} \bigr] \bigl\vert \psi''( \mu) \bigr\vert \,d\mu. \end{aligned} $$

Since $(b_{2}-b_{1})^{\alpha}(b_{2}-\mu)-(b_{2}-\mu)^{\alpha+1}\geq0$ and $|\psi''|$ is an increasing function, therefore we have

$$\begin{aligned} & \biggl\vert \frac{\alpha\psi(b_{1})+\psi(b_{2})}{\alpha+1}-\frac{\Gamma(\alpha+1)}{(b_{2}-b_{1})^{\alpha}}J_{b_{1}+}^{\alpha} \psi(b_{2}) \biggr\vert \\ &\quad \leq\frac{ \vert \psi''(b_{2}) \vert }{(\alpha+1)(b_{2}-b_{1})^{\alpha}} \int_{b_{1}}^{b_{2}} \bigl[(b_{2}-b_{1})^{\alpha}(b_{2}- \mu)-(b_{2}-\mu)^{\alpha+1} \bigr]\,d\mu \\ &\quad =\frac{ \vert \psi''(b_{2}) \vert }{(\alpha+1)(b_{2}-b_{1})^{\alpha}} \biggl[(b_{2}-b_{1})^{\alpha}\frac{(b_{2}-b_{1})^{2}}{2}-\frac{(b_{2}-b_{1})^{\alpha+2}}{\alpha+2} \biggr] \\ &\quad =\frac{ \vert \psi''(b_{2}) \vert \alpha(b_{2}-b_{1})^{2}}{2(\alpha+1)(\alpha+2)}, \end{aligned}$$

which is inequality (2.17).

Part (ii) can be proved in a similar way, we omit the details.

For part (iii), making use of (2.18) and the fact that every convex function *ψ* defined on the interval $[b_{1},b_{2}]$ is bounded above by $\max\{\psi(b_{1}),\psi(b_{2})\}$, we get

$$\begin{aligned} & \biggl\vert \frac{\alpha\psi(b_{1})+\psi(b_{2})}{\alpha+1}-\frac{\Gamma(\alpha+1)}{(b_{2}-b_{1})^{\alpha}}J_{b_{1}+}^{\alpha} \psi(b_{2}) \biggr\vert \\ &\quad \leq\frac{\max\{ \vert \psi''(b_{1}) \vert , \vert \psi''(b_{2}) \vert \}}{(\alpha+1)(b_{2}-b_{1})^{\alpha}} \int_{b_{1}}^{b_{2}} \bigl[(b_{2}-b_{1})^{\alpha}(b_{2}- \mu)-(b_{2}-\mu)^{\alpha+1} \bigr]\,d\mu \\ &\quad =\frac{\max\{ \vert \psi''(b_{1}) \vert , \vert \psi''(b_{2}) \vert \}\alpha(b_{2}-b_{1})^{2}}{2(\alpha+1)(\alpha+2)}. \end{aligned}$$

 □

### Remark 2.4

Let $\alpha=1$. Then Theorem 2.3 leads to

$$\begin{aligned} & \biggl\vert \frac{\psi(b_{1})+\psi(b_{2})}{2}-\frac{1}{b_{2}-b_{1}} \int_{b_{1}}^{b_{2}}\psi(t)\,dt \biggr\vert \leq \frac{ \vert \psi''(b_{2}) \vert (b_{2}-b_{1})^{2}}{12}, \\ & \biggl\vert \frac{\psi(b_{1})+\psi(b_{2})}{2}-\frac{1}{b_{2}-b_{1}} \int_{b_{1}}^{b_{2}}\psi(t)\,dt \biggr\vert \leq \frac{ \vert \psi''(b_{1}) \vert (b_{2}-b_{1})^{2}}{12}, \\ & \biggl\vert \frac{\psi(b_{1})+\psi(b_{2})}{2}-\frac{1}{b_{2}-b_{1}} \int_{b_{1}}^{b_{2}}\psi(t)\,dt \biggr\vert \leq \frac{\max\{ \vert \psi''(b_{1}) \vert , \vert \psi''(b_{2}) \vert \}(b_{2}-b_{1})^{2}}{12}. \end{aligned}$$

### Theorem 2.5

*Let* $\psi \in C^{2}([b_{1},b_{2}])$ *and* $\alpha>0$. *Then the following statements are true*:

(i) *If* $|\psi''|$ *is an increasing function*, *then*
  $$\begin{aligned} & \biggl\vert \psi \biggl(\frac{\alpha b_{1}+b_{2}}{\alpha+1} \biggr)-\frac{\Gamma(\alpha+1)}{(b_{2}-b_{1})^{\alpha}}J_{b_{1}+}^{\alpha} \psi(b_{2}) \biggr\vert \\ &\quad \leq\frac{(b_{2}-b_{1})^{2}}{(\alpha+1)^{\alpha+3}(\alpha+2)} \\ &\qquad {}\times\biggl[ \biggl\vert \psi'' \biggl(\frac{\alpha b_{1}+b_{2}}{\alpha+1} \biggr) \biggr\vert \biggl(\frac{\alpha[(\alpha+1)^{\alpha+1}-2\alpha^{\alpha+1}]}{2} \biggr)+ \bigl\vert \psi''(b_{2}) \bigr\vert \alpha^{\alpha+2} \biggr]. \end{aligned}$$
(ii) *If* $|\psi''|$ *is a decreasing function*, *then*
  $$\begin{aligned} &\biggl\vert \psi \biggl(\frac{\alpha b_{1}+b_{2}}{\alpha+1} \biggr)-\frac{\Gamma(\alpha+1)}{(b_{2}-b_{1})^{\alpha}}J_{b_{1}+}^{\alpha} \psi(b_{2}) \biggr\vert \\ &\quad \leq\frac{(b_{2}-b_{1})^{2}}{(\alpha+1)^{\alpha+3}(\alpha+2)} \\ &\qquad {}\times\biggl[ \bigl\vert \psi''(b_{1}) \bigr\vert \biggl(\frac{\alpha[(\alpha+1)^{\alpha+1}-2\alpha^{\alpha+1}]}{2} \biggr)+ \biggl\vert \psi'' \biggl(\frac{\alpha b_{1}+b_{2}}{\alpha+1} \biggr) \biggr\vert \alpha^{\alpha+2} \biggr]. \end{aligned}$$
(iii) *If* $|\psi''|$ *is a convex function*, *then*
  $$\begin{aligned} &\biggl\vert \psi \biggl(\frac{\alpha b_{1}+b_{2}}{\alpha+1} \biggr)-\frac{\Gamma(\alpha+1)}{(b_{2}-b_{1})^{\alpha}}J_{b_{1}+}^{\alpha} \psi(b_{2}) \biggr\vert \\ &\quad \leq\frac{(b_{2}-b_{1})^{2}}{(\alpha+1)^{\alpha+3}(\alpha+2)} \\ &\qquad {}\times \biggl[\max \biggl\{ \bigl\vert \psi''(b_{1}) \bigr\vert , \biggl\vert \psi'' \biggl( \frac{\alpha b_{1}+b_{2}}{\alpha+1} \biggr) \biggr\vert \biggr\} \biggl(\frac{\alpha[(\alpha+1)^{\alpha+1}-2\alpha^{\alpha+1}]}{2} \biggr) \\ &\qquad {}+\max \biggl\{ \biggl\vert \psi'' \biggl( \frac{\alpha b_{1}+b_{2}}{\alpha+1} \biggr) \biggr\vert , \bigl\vert \psi''(b_{2}) \bigr\vert \biggr\} \alpha^{\alpha+2} \biggr]. \end{aligned}$$

### Proof

(i) It follows from (2.9) that

2.19 $$\begin{aligned} &\psi \biggl(\frac{\alpha b_{1}+b_{2}}{\alpha+1} \biggr)- \frac{\Gamma(\alpha+1)}{(b_{2}-b_{1})^{\alpha}}J_{b_{1}+}^{\alpha} \psi(b_{2}) \\ &\quad = \int_{b_{1}}^{\frac{\alpha b_{1}+b_{2}}{\alpha+1}} \biggl[G \biggl(\frac{\alpha b_{1}+b_{2}}{\alpha+1},\mu \biggr)-\frac{\alpha}{(b_{2}-b_{1})^{\alpha}} \int_{b_{1}}^{b_{2}}(b_{2}-x)^{\alpha-1}G(x, \mu)\,dx \biggr]\psi''(\mu) \,d\mu \\ &\qquad {}+ \int_{\frac{\alpha b_{1}+b_{2}}{\alpha+1}}^{b_{2}} \biggl[G \biggl(\frac{\alpha b_{1}+b_{2}}{\alpha+1},\mu \biggr)-\frac{\alpha}{(b_{2}-b_{1})^{\alpha}} \int_{b_{1}}^{b_{2}}(b_{2}-x)^{\alpha-1}G(x, \mu)\,dx \biggr]\psi''(\mu) \,d\mu \\ &\quad =-\frac{1}{(\alpha+1)(b_{2}-b_{1})^{\alpha}} \biggl[ \int_{b_{1}}^{\frac{\alpha b_{1}+b_{2}}{\alpha+1}} \bigl\{ (b_{2}- \mu)^{\alpha+1} -(b_{2}-b_{1})^{\alpha+1} \\ &\qquad {}-(b_{1}-\mu) (b_{2}-b_{1})^{\alpha}( \alpha+1) \bigr\} \psi''(\mu)\,d\mu + \int_{\frac{\alpha b_{1}+b_{2}}{\alpha+1}}^{b_{2}}(b_{2}-\mu)^{\alpha+1} \psi''(\mu)\,d\mu \biggr]. \end{aligned}$$

Taking the absolute function and using the triangular inequality, we get

$$\begin{aligned} & \biggl\vert \psi \biggl(\frac{\alpha b_{1}+b_{2}}{\alpha+1} \biggr)-\frac{\Gamma(\alpha+1)}{(b_{2}-b_{1})^{\alpha}}J_{b_{1}+}^{\alpha} \psi(b_{2}) \biggr\vert \\ &\quad \leq\frac{1}{(\alpha+1)(b_{2}-b_{1})^{\alpha}} \biggl[ \int_{b_{1}}^{\frac{\alpha b_{1}+b_{2}}{\alpha+1}} \bigl\{ (b_{2}- \mu)^{\alpha+1}-(b_{2}-b_{1})^{\alpha+1} \\ &\qquad {}-(b_{1}-\mu) (b_{2}-b_{1})^{\alpha}( \alpha+1) \bigr\} \bigl\vert \psi''(\mu) \bigr\vert \,d \mu + \int_{\frac{\alpha b_{1}+b_{2}}{\alpha+1}}^{b_{2}}(b_{2}-\mu)^{\alpha+1} \bigl\vert \psi''(\mu) \bigr\vert \,d\mu \biggr] \\ &\quad \leq\frac{1}{(\alpha+1)(b_{2}-b_{1})^{\alpha}} \biggl[ \biggl\vert \psi'' \biggl( \frac{\alpha b_{1}+b_{2}}{\alpha+1} \biggr) \biggr\vert \int_{b_{1}}^{\frac{\alpha b_{1}+b_{2}}{\alpha+1}} \bigl\{ (b_{2}- \mu)^{\alpha+1}-(b_{2}-b_{1})^{\alpha+1} \\ &\qquad {}-(b_{1}-\mu) (b_{2}-b_{1})^{\alpha}( \alpha+1) \bigr\} \,d\mu+ \bigl\vert \psi''(b_{2}) \bigr\vert \int_{\frac{\alpha b_{1}+b_{2}}{\alpha+1}}^{b_{2}}(b_{2}-\mu)^{\alpha+1}\,d \mu \biggr] \\ &\quad =\frac{1}{(\alpha+1)(b_{2}-b_{1})^{\alpha}} \biggl[ \biggl\vert \psi'' \biggl( \frac{\alpha b_{1}+b_{2}}{\alpha+1} \biggr) \biggr\vert (b_{2}-b_{1})^{\alpha+2} \biggl\{ \frac{-\alpha^{\alpha+2}}{(\alpha+1)^{\alpha+2}(\alpha+2)} \\ &\qquad {}+\frac{1}{(\alpha+2)} -\frac{1}{(\alpha+1)}+\frac{1}{2(\alpha+1)} \biggr\} + \bigl\vert \psi''(b_{2}) \bigr\vert (b_{2}-b_{1})^{\alpha+2} \biggl\{ \frac{\alpha^{\alpha+2}}{(\alpha+1)^{\alpha+2}(\alpha+2)} \biggr\} \biggr] \\ &\quad =\frac{(b_{2}-b_{1})^{2}}{(\alpha+1)^{\alpha+3}(\alpha+2)} \biggl[ \biggl\vert \psi'' \biggl(\frac{\alpha b_{1}+b_{2}}{\alpha+1} \biggr) \biggr\vert \biggl\{ \frac{\alpha[(\alpha+1)^{\alpha+1}-2\alpha^{\alpha+1}]}{2} \biggr\} + \bigl\vert \psi''(b_{2}) \bigr\vert \alpha^{\alpha+2} \biggr]. \end{aligned}$$

Part (ii) can be proved by using the same procedure.

Next, we prove part (iii). We clearly see that

$$\begin{aligned} & \biggl\vert \psi \biggl(\frac{\alpha b_{1}+b_{2}}{\alpha+1} \biggr)-\frac{\Gamma(\alpha+1)}{(b_{2}-b_{1})^{\alpha}}J_{b_{1}+}^{\alpha} \psi(b_{2}) \biggr\vert \\ &\quad \leq\frac{1}{(\alpha+1)(b_{2}-b_{1})^{\alpha}} \biggl[ \int_{b_{1}}^{\frac{\alpha b_{1}+b_{2}}{\alpha+1}} \bigl\{ (b_{2}- \mu)^{\alpha+1} -(b_{2}-b_{1})^{\alpha+1} \\ &\qquad {}-(b_{1}-\mu) (b_{2}-b_{1})^{\alpha}( \alpha+1) \bigr\} \bigl\vert \psi''(\mu) \bigr\vert \,d \mu + \int_{\frac{\alpha b_{1}+b_{2}}{\alpha+1}}^{b_{2}}(b_{2}-\mu)^{\alpha+1} \bigl\vert \psi''(\mu) \bigr\vert \,d\mu \biggr]. \end{aligned}$$

Since every convex function *ψ* defined on an interval $[b_{1},b_{2}]$ is bounded above by $\max\{\psi(b_{1}),\psi(b_{2})\}$. Therefore, we have

$$\begin{aligned} & \biggl\vert \psi \biggl(\frac{\alpha b_{1}+b_{2}}{\alpha+1} \biggr)-\frac{\Gamma(\alpha+1)}{(b_{2}-b_{1})^{\alpha}}J_{b_{1}+}^{\alpha} \psi(b_{2}) \biggr\vert \\ &\quad \leq\frac{1}{(\alpha+1)(b_{2}-b_{1})^{\alpha}} \biggl[\max \biggl\{ \bigl\vert \psi''(b_{1}) \bigr\vert , \biggl\vert \psi'' \biggl( \frac{\alpha b_{1}+b_{2}}{\alpha+1} \biggr) \biggr\vert \biggr\} , \\ & \int_{b_{1}}^{\frac{\alpha b_{1}+b_{2}}{\alpha+1}} \bigl\{ (b_{2}- \mu)^{\alpha+1}-(b_{2}-b_{1})^{\alpha+1}-(b_{1}- \mu) (b_{2}-b_{1})^{\alpha}(\alpha+1) \bigr\} \,d\mu \\ &\qquad {}+\max \biggl\{ \biggl\vert \psi'' \biggl( \frac{\alpha b_{1}+b_{2}}{\alpha+1} \biggr) \biggr\vert , \bigl\vert \psi''(b_{2}) \bigr\vert \biggr\} \int_{\frac{\alpha b_{1}+b_{2}}{\alpha+1}}^{b_{2}}(b_{2}-\mu)^{\alpha+1}\,d \mu \biggr] \\ &\quad =\frac{(b_{2}-b_{1})^{2}}{(\alpha+1)^{\alpha+3}(\alpha+2)} \biggl[\max \biggl\{ \bigl\vert \psi''(b_{1}) \bigr\vert , \biggl\vert \psi'' \biggl( \frac{\alpha b_{1}+b_{2}}{\alpha+1} \biggr) \biggr\vert \biggr\} \biggl(\frac{\alpha[(\alpha+1)^{\alpha+1}-2\alpha^{\alpha+1}]}{2} \biggr) \\ & \qquad {}+\max \biggl\{ \biggl\vert \psi'' \biggl(\frac{\alpha b_{1}+b_{2}}{\alpha+1} \biggr) \biggr\vert , \bigl\vert \psi''(b_{2}) \bigr\vert \biggr\} \bigl(\alpha^{\alpha+2} \bigr) \biggr], \end{aligned}$$

which is our required inequality. □

### Remark 2.6

Let $\alpha=1$. Then Theorem 2.5 leads to

$$\begin{aligned} & \biggl\vert \psi \biggl(\frac{b_{1}+b_{2}}{2} \biggr)-\frac{1}{b_{2}-b_{1}} \int_{b_{1}}^{b_{2}}\psi(t)\,dt \biggr\vert \leq \frac{(b_{2}-b_{1})^{2}}{48} \biggl[ \biggl\vert \psi'' \biggl( \frac{b_{1}+b_{2}}{2} \biggr) \biggr\vert + \bigl\vert \psi''(b_{2}) \bigr\vert \biggr], \\ & \biggl\vert \psi \biggl(\frac{b_{1}+b_{2}}{2} \biggr)-\frac{1}{b_{2}-b_{1}} \int_{b_{1}}^{b_{2}}\psi(t)\,dt \biggr\vert \leq \frac{(b_{2}-b_{1})^{2}}{48} \biggl[ \bigl\vert \psi''(b_{1}) \bigr\vert + \biggl\vert \psi'' \biggl( \frac{b_{1}+b_{2}}{2} \biggr) \biggr\vert \biggr], \\ & \biggl\vert \psi \biggl(\frac{b_{1}+b_{2}}{2} \biggr)-\frac{1}{b_{2}-b_{1}} \int_{b_{1}}^{b_{2}}\psi(t)\,dt \biggr\vert \\ &\quad \leq\frac{(b_{2}-b_{1})^{2}}{48} \biggl[\max \biggl\{ \bigl\vert \psi''(b_{1}) \bigr\vert , \biggl\vert \psi'' \biggl( \frac{b_{1}+b_{2}}{2} \biggr) \biggr\vert \biggr\} +\max \biggl\{ \biggl\vert \psi'' \biggl( \frac{b_{1}+b_{2}}{2} \biggr) \biggr\vert , \bigl\vert \psi''(b_{2}) \bigr\vert \biggr\} \biggr]. \end{aligned}$$

### Theorem 2.7

*Let* $\psi \in C^{2}([b_{1},b_{2}])$ *and* $|\psi''|$ *be a convex function*. *Then the inequality*

$$\begin{aligned} & \biggl\vert \psi \biggl(\frac{\alpha b_{1}+b_{2}}{\alpha+1} \biggr)-\frac{\Gamma(\alpha+1)}{(b_{2}-b_{1})^{\alpha}}J_{b_{1}+}^{\alpha} \psi(b_{2}) \biggr\vert \\ &\quad \leq\frac{(b_{2}-b_{1})^{2}}{6(\alpha+1)^{3}(\alpha+3)} \biggl[ \bigl\vert \psi''(b_{1}) \bigr\vert \bigl(9\alpha^{2}+23\alpha+12 \bigr)+\frac{ \vert \psi''(b_{2}) \vert (7\alpha^{2}+17\alpha+12)}{\alpha+2} \biggr] \end{aligned}$$

*holds for any* $\alpha>0$.

### Proof

It follows from (2.19) that

$$\begin{aligned} &\psi \biggl(\frac{\alpha b_{1}+b_{2}}{\alpha+1} \biggr)-\frac{\Gamma(\alpha+1)}{(b_{2}-b_{1})^{\alpha}}J_{b_{1}+}^{\alpha} \psi(b_{2}) \\ &\quad =\frac{1}{(\alpha+1)(b_{2}-b_{1})^{\alpha}} \biggl[ \int_{b_{1}}^{\frac{\alpha b_{1}+b_{2}}{\alpha+1}} \bigl\{ (b_{1}-\mu) (b_{2}-b_{1})^{\alpha}(\alpha+1) \\ &\qquad {}-(b_{2}-\mu)^{\alpha+1} +(b_{2}-b_{1})^{\alpha+1} \bigr\} \psi''(\mu)\,d\mu - \int_{\frac{\alpha b_{1}+b_{2}}{\alpha+1}}^{b_{2}}(b_{2}-\mu)^{\alpha+1} \psi''(\mu)\,d\mu \biggr]. \end{aligned}$$

Let $t\in[0,1]$ and $\mu=tb_{1}+(1-t)b_{2}$. Then

2.20 $$ \begin{aligned}[b] &\psi \biggl(\frac{\alpha b_{1}+b_{2}}{\alpha+1} \biggr)- \frac{\Gamma(\alpha+1)}{(b_{2}-b_{1})^{\alpha}}J_{b_{1}+}^{\alpha} \psi(b_{2}) \\ &\quad =\frac{1}{(\alpha+1)(b_{2}-b_{1})^{\alpha}} \biggl[ \int_{1}^{\frac{\alpha}{\alpha+1}} \bigl\{ \bigl[b_{1}-tb_{1}-(1-t)b_{2} \bigr](b_{2}-b_{1})^{\alpha}(\alpha+1) \\ &\qquad {}- \bigl[b_{2}-tb_{1}-(1-t)b_{2} \bigr]^{\alpha+1}+(b_{2}-b_{1})^{\alpha+1} \bigr\} \psi'' \bigl(tb_{1}+(1-t)b_{2} \bigr) (b_{1}-b_{2})\,dt \\ &\qquad {}- \int_{\frac{\alpha}{\alpha+1}}^{0} \bigl[b_{2}-tb_{1}-(1-t)b_{2} \bigr]^{\alpha+1}\psi'' \bigl(tb_{1}+(1-t)b_{2} \bigr) (b_{1}-b_{2})\,dt \biggr] \\ &\quad =\frac{-(b_{2}-b_{1})^{\alpha+2}}{(\alpha+1)(b_{2}-b_{1})^{\alpha}} \biggl[ \int_{1}^{\frac{\alpha}{\alpha+1}} \bigl\{ (-1+t) (\alpha+1)+1 \bigr\} \psi'' \bigl(tb_{1}+(1-t)b_{2} \bigr) \,dt \\ &\qquad {}- \int_{1}^{\frac{\alpha}{\alpha+1}}t^{\alpha+1}\psi'' \bigl(tb_{1}+(1-t)b_{2} \bigr)\,dt+ \int_{0}^{\frac{\alpha}{\alpha+1}}t^{\alpha+1}\psi'' \bigl(tb_{1}+(1-t)b_{2} \bigr)\,dt \biggr] \\ &\quad =\frac{(b_{2}-b_{1})^{2}}{\alpha+1} \biggl[ \int_{\frac{\alpha}{\alpha+1}}^{1}(\alpha t+t-\alpha) \psi'' \bigl(tb_{1}+(1-t)b_{2} \bigr) \,dt\\ &\qquad {} - \int_{0}^{1}t^{\alpha+1}\psi'' \bigl(tb_{1}+(1-t)b_{2} \bigr)\,dt \biggr]. \end{aligned} $$

Taking absolute on both sides and using the convexity of $|\psi''|$, we get

$$\begin{aligned} & \biggl\vert \psi \biggl(\frac{\alpha b_{1}+b_{2}}{\alpha+1} \biggr)-\frac{\Gamma(\alpha+1)}{(b_{2}-b_{1})^{\alpha}}J_{b_{1}+}^{\alpha} \psi(b_{2}) \biggr\vert \\ &\quad \leq\frac{(b_{2}-b_{1})^{2}}{\alpha+1} \biggl[ \int_{\frac{\alpha}{\alpha+1}}^{1}(\alpha t+t-\alpha) \bigl\vert \psi'' \bigl(tb_{1}+(1-t)b_{2} \bigr) \bigr\vert \,dt\\ &\qquad {} + \int_{0}^{1}t^{\alpha+1} \bigl\vert \psi'' \bigl(tb_{1}+(1-t)b_{2} \bigr) \bigr\vert \,dt \biggr] \\ &\quad \leq\frac{(b_{2}-b_{1})^{2}}{\alpha+1} \biggl[ \int_{\frac{\alpha}{\alpha+1}}^{1} \bigl(t(\alpha+1)-\alpha \bigr) \bigl[t \bigl\vert \psi''(b_{1}) \bigr\vert +(1-t) \bigl\vert \psi''(b_{2}) \bigr\vert \bigr]\,dt \\ &\qquad {}+ \int_{0}^{1}t^{\alpha+1} \bigl[t \bigl\vert \psi''(b_{1}) \bigr\vert +(1-t) \bigl\vert \psi''(b_{2}) \bigr\vert \bigr]\,dt \biggr] \\ &\quad =\frac{(b_{2}-b_{1})^{2}}{\alpha+1} \biggl[\frac{ \vert \psi''(b_{1}) \vert (3\alpha+2)}{6(\alpha+1)^{2}}+\frac{ \vert \psi''(b_{2}) \vert }{6(\alpha+1)^{2}} + \frac{ \vert \psi''(b_{1}) \vert }{\alpha+3}+\frac{ \vert \psi''(b_{2}) \vert }{(\alpha+2)(\alpha+3)} \biggr] \\ &\quad =\frac{(b_{2}-b_{1})^{2}}{6(\alpha+1)^{3}(\alpha+3)} \biggl[ \bigl\vert \psi''(b_{1}) \bigr\vert \bigl(9\alpha^{2}+23\alpha+12 \bigr)+\frac{ \vert \psi''(b_{2}) \vert (7\alpha^{2}+17\alpha+12)}{\alpha+2} \biggr], \end{aligned}$$

which completes the proof. □

### Remark 2.8

In Theorem 2.7, if we take $\alpha=1$, then we obtain

$$ \biggl\vert \psi \biggl(\frac{b_{1}+b_{2}}{2} \biggr)-\frac{1}{b_{2}-b_{1}} \int_{b_{1}}^{b_{2}}\psi(t)\,dt \biggr\vert \leq \frac{(b_{2}-b_{1})^{2}}{48} \bigl[11|\psi''(b_{1})+3 \bigl\vert \psi''(b_{2}) \bigr\vert \bigr]. $$

### Theorem 2.9

*Let* $\psi \in C^{2}([b_{1},b_{2}])$ *and* $|\psi''|$ *be a convex function*. *Then the inequality*

$$\begin{aligned} & \biggl\vert \frac{\alpha\psi(b_{1})+\psi(b_{2})}{\alpha+1}-\frac{\Gamma(\alpha+1)}{(b_{2}-b_{1})^{\alpha}}J_{b_{1}+}^{\alpha} \psi(b_{2}) \biggr\vert \\ &\quad \leq\frac{\alpha(b_{2}-b_{1})^{2}}{3(\alpha+1)(\alpha+3)} \biggl[ \bigl\vert \psi''(b_{1}) \bigr\vert + \bigl\vert \psi''(b_{2}) \bigr\vert \biggl(\frac{\alpha+5}{2(\alpha+2)} \biggr) \biggr] \end{aligned}$$

*holds for any* $\alpha>0$.

### Proof

From (2.15), we have

$$\begin{aligned} &\frac{\alpha\psi(b_{1})+\psi(b_{2})}{\alpha+1}-\frac{\Gamma(\alpha+1)}{(b_{2}-b_{1})^{\alpha}}J_{b_{1}+}^{\alpha} \psi(b_{2}) \\ &\quad =\frac{1}{(\alpha+1)(b_{2}-b_{1})^{\alpha}} \int_{b_{1}}^{b_{2}} \bigl[(b_{2}-b_{1})^{\alpha}(b_{2}- \mu)-(b_{2}-\mu)^{\alpha+1} \bigr]\psi''( \mu)\,d\mu. \end{aligned}$$

Let $t\in[0,1]$ and $\mu=tb_{1}+(1-t)b_{2}$. Then we obtain

2.21 $$ \begin{aligned}[b] &\frac{\alpha\psi(b_{1})+\psi(b_{2})}{\alpha+1}-\frac{\Gamma(\alpha+1)}{(b_{2}-b_{1})^{\alpha}}J_{b_{1}+}^{\alpha} \psi(b_{2}) \\ &\quad =\frac{b_{2}-b_{1}}{(\alpha+1)(b_{2}-b_{1})^{\alpha}}\\ &\qquad {}\times \int_{0}^{1} \bigl\{ (b_{2}-b_{1})^{\alpha}\bigl[t(b_{2}-b_{1}) \bigr]- \bigl[t(b_{2}-b_{1}) \bigr]^{\alpha+1} \bigr\} \psi'' \bigl(tb_{1}+(1-t)b_{2} \bigr)\,dt \\ &\quad =\frac{(b_{2}-b_{1})^{2}}{\alpha+1} \int_{0}^{1} \bigl(t-t^{\alpha+1} \bigr) \psi'' \bigl(tb_{1}+(1-t)b_{2} \bigr) \,dt. \end{aligned} $$

Taking absolute on both sides and using the convexity of $|\psi''|$, we get

$$\begin{aligned} & \biggl\vert \frac{\alpha\psi(b_{1})+\psi(b_{2})}{\alpha+1}-\frac{\Gamma(\alpha+1)}{(b_{2}-b_{1})^{\alpha}}J_{b_{1}+}^{\alpha} \psi(b_{2}) \biggr\vert \\ &\quad \leq\frac{(b_{2}-b_{1})^{2}}{\alpha+1} \int_{0}^{1} \bigl(t-t^{\alpha+1} \bigr) \bigl\vert \psi'' \bigl(tb_{1}+(1-t)b_{2} \bigr) \bigr\vert \,dt \\ &\quad \leq\frac{(b_{2}-b_{1})^{2}}{\alpha+1} \int_{0}^{1} \bigl(t-t^{\alpha+1} \bigr) \bigl[t \bigl\vert \psi''(b_{1}) \bigr\vert +(1-t) \bigl\vert \psi''(b_{2}) \bigr\vert \bigr]\,dt \\ &\quad =\frac{(b_{2}-b_{1})^{2}}{\alpha+1} \biggl[ \bigl\vert \psi''(b_{1}) \bigr\vert \biggl(\frac{\alpha}{3(\alpha+3)} \biggr) + \bigl\vert \psi''(b_{2}) \bigr\vert \biggl( \frac{\alpha^{2}+5\alpha}{6(\alpha+2)(\alpha+3)} \biggr) \biggr] \\ &\quad =\frac{\alpha(b_{2}-b_{1})^{2}}{3(\alpha+1)(\alpha+3)} \biggl[ \bigl\vert \psi''(b_{1}) \bigr\vert + \bigl\vert \psi''(b_{2}) \bigr\vert \biggl(\frac{\alpha+5}{2(\alpha+2)} \biggr) \biggr], \end{aligned}$$

which is our required inequality. □

### Remark 2.10

In Theorem 2.9, if we take $\alpha=1$, then we obtain

$$ \biggl\vert \frac{\psi(b_{1})+\psi(b_{2})}{2}-\frac{1}{b_{2}-b_{1}} \int_{b_{1}}^{b_{2}}\psi(t)\,dt \biggr\vert \leq \frac{(b_{2}-b_{1})^{2}}{24} \bigl[ \bigl\vert \psi''(b_{1})+| \psi''(b_{2}) \bigr\vert \bigr]. $$

### Theorem 2.11

*Let* $\psi \in C^{2}([b_{1},b_{2}])$ *and* $|\psi''|$ *be a concave function*. *Then*

$$\begin{aligned} & \biggl\vert \psi \biggl(\frac{\alpha b_{1}+b_{2}}{\alpha+1} \biggr)-\frac{\Gamma(\alpha+1)}{(b_{2}-b_{1})^{\alpha}}J_{b_{1}+}^{\alpha} \psi(b_{2}) \biggr\vert \\ &\quad \leq\frac{(b_{2}-b_{1})^{2}}{\alpha+1} \biggl[\frac{1}{2(\alpha+1)} \biggl\vert \psi'' \biggl(\frac{3\alpha b_{1}+2b_{1}+b_{2}}{3(\alpha+1)} \biggr) \biggr\vert + \frac{1}{\alpha+2} \biggl\vert \psi'' \biggl( \frac{\alpha b_{1}+2b_{1}+b_{2}}{\alpha+3} \biggr) \biggr\vert \biggr] \end{aligned}$$

*for any* $\alpha>0$.

### Proof

It follows from (2.20) that

$$\begin{aligned} &\psi \biggl(\frac{\alpha b_{1}+b_{2}}{\alpha+1} \biggr)-\frac{\Gamma(\alpha+1)}{(b_{2}-b_{1})^{\alpha}}J_{b_{1}+}^{\alpha} \psi(b_{2}) \\ &\quad =\frac{(b_{2}-b_{1})^{2}}{\alpha+1}\\ &\qquad {}\times \biggl[ \int_{\frac{\alpha}{\alpha+1}}^{1} \bigl\{ t(\alpha+1)-\alpha \bigr\} \psi'' \bigl(tb_{1}+(1-t)b_{2} \bigr) \,dt - \int_{0}^{1}t^{\alpha+1}\psi'' \bigl(tb_{1}+(1-t)b_{2} \bigr)\,dt \biggr]. \end{aligned}$$

Taking absolute on both sides and using Jensen’s integral inequality, we get

$$\begin{aligned} & \biggl\vert \psi \biggl(\frac{\alpha b_{1}+b_{2}}{\alpha+1} \biggr)-\frac{\Gamma(\alpha+1)}{(b_{2}-b_{1})^{\alpha}}J_{b_{1}+}^{\alpha} \psi(b_{2}) \biggr\vert \\ &\quad \leq\frac{(b_{2}-b_{1})^{2}}{\alpha+1} \biggl[ \int_{\frac{\alpha}{\alpha+1}}^{1} \bigl\{ t(\alpha+1)-\alpha \bigr\} \,dt \biggl\vert \psi'' \biggl(\frac{\int_{\frac{\alpha}{\alpha+1}}^{1}\{t(\alpha+1)-\alpha\}\{tb_{1}+(1-t)b_{2}\}\,dt}{ \int_{\frac{\alpha}{\alpha+1}}^{1}\{t(\alpha+1)-\alpha\}\,dt} \biggr) \biggr\vert \\ &\qquad {}+ \int_{0}^{1}t^{\alpha+1}\,dt \biggl\vert \psi'' \biggl(\frac{\int_{0}^{1}t^{\alpha+1}\{tb_{1}+(1-t)b_{2}\}\,dt}{\int_{0}^{1}t^{\alpha+1}\,dt} \biggr) \biggr\vert \biggr] \\ &\quad =\frac{(b_{2}-b_{1})^{2}}{\alpha+1} \biggl[\frac{1}{2(\alpha+1)} \biggl\vert \psi'' \biggl(\frac{3\alpha b_{1}+2b_{1}+b_{2}}{3(\alpha+1)} \biggr) \biggr\vert + \frac{1}{\alpha+2} \biggl\vert \psi'' \biggl( \frac{\alpha b_{1}+2b_{1}+b_{2}}{\alpha+3} \biggr) \biggr\vert \biggr]. \end{aligned}$$

 □

### Remark 2.12

In Theorem 2.11, if we take $\alpha=1$, then we obtain

$$\begin{aligned} &\biggl\vert \psi \biggl(\frac{b_{1}+b_{2}}{2} \biggr)-\frac{1}{b_{2}-b_{1}} \int_{b_{1}}^{b_{2}}\psi(t)\,dt \biggr\vert \\ &\quad \leq \frac{(b_{2}-b_{1})^{2}}{2} \biggl[\frac{1}{4} \biggl\vert \psi'' \biggl(\frac{5b_{1}+b_{2}}{6} \biggr) \biggr\vert +\frac{1}{3} \biggl\vert \psi'' \biggl(\frac{3b_{1}+b_{2}}{4} \biggr) \biggr\vert \biggr]. \end{aligned}$$

### Theorem 2.13

*Assume that* $\psi \in C^{2}([b_{1},b_{2}])$ *and* $|\psi''|$ *is a concave function*. *Then*, *for any* $\alpha>0$, *we have the inequality*

$$\begin{aligned} & \biggl\vert \frac{\alpha\psi(b_{1})+\psi(b_{2})}{\alpha+1}-\frac{\Gamma(\alpha+1)}{(b_{2}-b_{1})^{\alpha}}J_{b_{1}+}^{\alpha} \psi(b_{2}) \biggr\vert \\ &\quad \leq\frac{\alpha(b_{2}-b_{1})^{2}}{2(\alpha+1)(\alpha+2)} \biggl\vert \psi'' \biggl( \frac{2\alpha b_{1}+\alpha b_{2}+4b_{1}+5b_{2}}{3(\alpha+3)} \biggr) \biggr\vert . \end{aligned}$$

### Proof

From (2.21) one has

$$\begin{aligned} &\frac{\alpha\psi(b_{1})+\psi(b_{2})}{\alpha+1}-\frac{\Gamma(\alpha+1)}{(b_{2}-b_{1})^{\alpha}}J_{b_{1}+}^{\alpha} \psi(b_{2}) \\ &\quad =\frac{(b_{2}-b_{1})^{2}}{\alpha+1} \int_{0}^{1} \bigl(t-t^{\alpha+1} \bigr) \psi'' \bigl(tb_{1}+(1-t)b_{2} \bigr) \,dt. \end{aligned}$$

Taking absolute on both sides and using Jensen’s integral inequality, we get

$$\begin{aligned} & \biggl\vert \frac{\alpha\psi(b_{1})+\psi(b_{2})}{\alpha+1}-\frac{\Gamma(\alpha+1)}{(b_{2}-b_{1})^{\alpha}}J_{b_{1}+}^{\alpha} \psi(b_{2}) \biggr\vert \\ &\quad \leq\frac{(b_{2}-b_{1})^{2}}{\alpha+1} \int_{0}^{1} \bigl(t-t^{\alpha+1} \bigr)\,dt \biggl\vert \psi'' \biggl(\frac{\int_{0}^{1} (t-t^{\alpha+1} )\{tb_{1}+(1-t)b_{2}\}\,dt}{\int_{0}^{1} (t-t^{\alpha+1} )\,dt} \biggr) \biggr\vert \\ &\quad =\frac{\alpha(b_{2}-b_{1})^{2}}{2(\alpha+1)(\alpha+2)} \biggl\vert \psi'' \biggl( \frac{2\alpha b_{1}+\alpha b_{2}+4b_{1}+5b_{2}}{3(\alpha+3)} \biggr) \biggr\vert . \end{aligned}$$

 □

### Remark 2.14

Let $\alpha=1$. Then Theorem 2.13 leads to

$$ \biggl\vert \frac{\psi(b_{1})+\psi(b_{2})}{2}-\frac{1}{b_{2}-b_{1}} \int_{b_{1}}^{b_{2}}\psi(t)\,dt \biggr\vert \leq \frac{(b_{2}-b_{1})^{2}}{12} \biggl\vert \psi'' \biggl( \frac{b_{1}+b_{2}}{2} \biggr) \biggr\vert . $$

### Remark 2.15

We can also get the same results as in this article if we use the Green’s function $G_{2}$ given by (2.2) instead of the Green’s function $G_{1}$ given by (2.1). But we can only get previously known results if we use the idea of the article and the Green’s function $G_{3}$ given by (2.3) or $G_{4}$ given by (2.4). This is the reason why we only deal with the Green’s function $G_{1}$ in the article. As exercises, interested readers can use other three Green’s functions to give their corresponding results.

## Results and discussion

In the article, we use Green’s function method to establish the left Riemann–Liouville fractional Hermite–Hadamard type inequalities. The given idea by using the Green’s function (2.1) or any other new Green’s function may be furthered to research the Hermite–Hadamard inequality for fractional integrals as presented in [38] and to research the Hermite–Hadamard inequality for pre-invex, *s*-convex, co-ordinate convex functions etc.

## Conclusion

In the article, we establish the left Riemann–Liouville fractional Hermite–Hadamard type inequalities and the generalized Hermite–Hadamard type inequalities by using Green’s function and Jensen’s inequality. The given idea and results are novel and interesting, they may stimulate further research in the theory of fractional integrals and generalized convex functions.
